# Pediatric Mental and Behavioral Health in the Period of Quarantine and Social Distancing With COVID-19

**DOI:** 10.2196/19867

**Published:** 2020-07-28

**Authors:** Jiancheng Ye

**Affiliations:** 1 Feinberg School of Medicine Northwestern University Chicago, IL United States

**Keywords:** pediatrics, mental health, stay-at-home orders, health technology, digital interventions, social distancing, COVID-19

## Abstract

The coronavirus disease (COVID-19) pandemic has spread rapidly throughout the world and has had a long-term impact. The pandemic has caused great harm to society and caused serious psychological trauma to many people. Children are a vulnerable group in this global public health emergency, as their nervous systems, endocrine systems, and hypothalamic-pituitary-adrenal axes are not well developed. Psychological crises often cause children to produce feelings of abandonment, despair, incapacity, and exhaustion, and even raise the risk of suicide. Children with mental illnesses are especially vulnerable during the quarantine and social distancing period. The inclusion of psychosocial support for children and their families are part of the health responses to disaster and disaster recovery. Based on the biopsychosocial model, some children may have catastrophic thoughts and be prone to experience despair, numbness, flashbacks, and other serious emotional and behavioral reactions. In severe cases, there may be symptoms of psychosis or posttraumatic stress disorder. Timely and appropriate protections are needed to prevent the occurrence of psychological and behavioral problems. The emerging digital applications and health services such as telehealth, social media, mobile health, and remote interactive online education are able to bridge the social distance and support mental and behavioral health for children. Based on the psychological development characteristics of children, this study also illustrates interventions on the psychological impact from the COVID-19 pandemic. Even though the world has been struggling to curb the influences of the pandemic, the quarantine and social distancing policies will have long-term impacts on children. Innovative digital solutions and informatics tools are needed more than ever to mitigate the negative consequences on children. Health care delivery and services should envision and implement innovative paradigms to meet broad well-being needs and child health as the quarantine and social distancing over a longer term becomes a new reality. Future research on children's mental and behavioral health should pay more attention to novel solutions that incorporate cutting edge interactive technologies and digital approaches, leveraging considerable advances in pervasive and ubiquitous computing, human-computer interaction, and health informatics among many others. Digital approaches, health technologies, and informatics are supposed to be designed and implemented to support public health surveillance and critical responses to children’s growth and development. For instance, human-computer interactions, augmented reality, and virtual reality could be incorporated to remote psychological supporting service for children’s health; mobile technologies could be used to monitor children’s mental and behavioral health while protecting their individual privacy; big data and artificial intelligence could be used to support decision making on whether children should go out for physical activities and whether schools should be reopened. Implications to clinical practices, psychological therapeutic practices, and future research directions to address current effort gaps are highlighted in this study.

## Introduction

Due to the spread of the coronavirus disease (COVID-19), people in many counties such as the United States, China, and Italy are restricted from leaving homes for anything other than essential activities [1-4]. The long-term home confinement has adverse effects on children’s physical and mental health to a certain degree [5]. Studies have shown that children who experienced quarantine are more likely to report high depressive and stress symptoms [6,7]. Longer duration of home confinement may result in poor mental health and avoidance behaviors [5]. Since children are not engaged in their “normal” class schedules, they may be experiencing fewer physical activities, irregular sleep rhythm and unhealthy diets, and longer smartphone screen exposure, resulting in physical problems such as increased body mass and decreased cardiopulmonary fitness [8]. Children’s mental and behavioral health are vulnerable to risks from the external environment, which will impact their development when they grow up [9]. Limited outdoor activities and lack of interaction with peers have a psychological impact on children as well. Lacking face-to-face contact with classmates and friends, and having a lack of personal space may also be detrimental to children’s overall health [5,8]. These negative influences have been reported to be as risky as other traumatic experiences [10], so it is warranted to address children’s mental and behavioral health.

## Psychogenic and Stress Reduction

Stress response [11] refers to the human body’s physical and mental response to an awareness of major changes or threats. Emotional states and clinical symptoms are influenced by the crisis, requiring psychological assistance and care. Anxiety and depression are common emotional reactions [12-14]. Specifically, preschool children may cry more and become clingy to others; school-aged children may be more nervous and scared, and repeatedly ask parents about the situation of the pandemic. Adolescents may have worries, irritability, and tantrums; some adolescents spend a lot of time watching the news about the pandemic [15]. Children of different ages may all experience poor appetite, insomnia, nightmares, etc [16,17].

## Psychological and Behavioral Changes Caused by Stress Response

Mild stress or chronic stress response is manifested as mild emotional, cognitive, and physical symptoms, which has little effect on daily life [18]. Moderate stress response can last for a few hours, and it affects physical, emotional, and cognitive functions [19]. There may be increased alertness, mainly manifested as being easily frightened, accompanied by inattention, increased irritability, and anxiety. Even slight sounds can result in children’s emotional instability and startled reactions. In this case, children need to seek professional psychological counseling for help. Severe stress [20] response seriously affects the life and learning abilities of children, which may lead to a series of psychotic manifestations [21]. If children have an emotional and behavioral stress reaction over 2 weeks, then they should go to the hospital for examination, diagnosis, and treatment, as well as psychiatric treatment as soon as possible to avoid prolonged illness [8].

Mild stress could increase attention, memory, and cognition in children, allowing them to adapt to changes in the external environment, which could be treated as a positive psychological response [22]. However, excessively intense stress can cause negative mental stress, such as dim consciousness; narrowed scope of consciousness; impaired attention; decreased memory, thinking, and imagination; and weakened learning ability [23]. Under stressful situations, children’s attitudes toward outside could also be distorted. The narrowed scope of cognitive ability may cause children to just focus on pandemic situations, disease, and other related negative contents, and no longer care about other positive things in the surrounding environment. They are likely to pay more attention to negative consequences or bad news. Only with the recovery of the psychological disorder and ceasing to worry about the crisis can most children gradually return to a normal thinking mode and cognitive state [24].

Along with psychological stress response, the children’s behaviors also seem to have changed [24]. This is the corresponding response that the body adopts to buffer the impact of stress and get rid of physical and mental tension, and adapt to the needs of the environment. Some children may have behavior inhibition reactions, such as a sense of loss and stupor, reduction of daily activities, unwillingness to communicate with others, and laziness in personal life [25]. They may also have anger, impatience, disobedience, and antagonism with families; experience interpersonal tension; and even behave with impulsive aggression [15].

For children who are infected, strict isolation in hospitals increases the distance between them and other people, which makes them feel helpless and desperate [26]. In these unfamiliar settings, children may not be able to control anxiety, cowardice, and stubbornness, thus showing resistance to treatment due to the environmental changes and fear of death. Some children with serious illness may even have symptoms such as a feeling of near-death, panic and despair, etc. Even when recovered, some children may continue to recall the details of the unpleasant experiences of being quarantined during the pandemic [27].

## The Roles of Communities and Schools

Communities, neighborhoods, and schools need to be aware of this negative impact on children and take timely and effective actions to deal with these problems [28]. Online interactive courses that provide a better learning experience can promote children’s healthy lifestyle while ensuring the content meets the educational needs without overburdening them. Communities or neighborhoods are usually important social resources to assist families and serve as a bridge between students and schools. Communities could invite psychologists to provide online services to cope with family conflicts, parent-child tensions, and mental health problems caused by concerns about the pandemic. Social workers in the neighborhoods play an active role in helping parents deal with family problems. This kind of social safety network is particularly useful for families in need or single-parent families.

## Parents Are the Closest Providers for Children

The main cause of panic during the public health crisis is that it destroys the daily life [29] that people are familiar with; in other words, it destroys the sense of security. The state of mental health is not only a bridge and link between the body and mind but also an important factor closely related to child immunity. Children’s stable emotions are the most powerful protection against viruses, so it is important to pay attention to children’s emotions and to manage, counsel, and intervene symptomatically.

With the premise of safety, parents should try to maintain children’s daily life rhythms such as work and rest balance and regular activities. Children should focus more on adequate daily activities such as reading, indoor sports, games, and handicrafts rather than paying too much attention to information about the pandemic. Entertainment activities can effectively relax their mind and brain. Parents should ensure that children have regular meals and nutrients, a comfortable family environment, and adequate sleep. Relieving psychological stress is the premise to ensuring a stable physiological state. Due to the COVID-19 pandemic, medical resources are limited to common mild diseases or chronic diseases. Meanwhile, going to health care settings may expose children to higher risks of getting infected. Point-of-care systems such as portable smart devices, [30] diagnosis technologies at home with the Internet of Things [31], and other digital interventions [32,33] play crucial roles to protect children during this period.

## Families Are the Frontline Psychological Counselors for Children

Parents are the first and best teachers for children, and good educational style becomes particularly important during the period of the pandemic. In addition to monitoring children’s performance and behavior, parents also need to respect children’s sense of identity and needs, and help them improve self-management capabilities. During the public health emergency, adolescents who have a certain degree of education will be exposed to a large amount of information related to the pandemic [24]. Parents should actively communicate with adolescents with effective ways to help them relieve anxiety and avoid panic. A good parenting style can strengthen family bonds and satisfy children’s psychological needs [34,35]. On the other hand, staying at home instead becomes a wonderful opportunity to strengthen parent-child relationships, facilitating children to actively participate in housework and improve abilities to take care of themselves.

During this special period, many parents are also prone to emotional instability and some of them even have quarrels and conflicts. These tensions and insecurity may be transmitted to the children with increased stress response [35]. Relieving stress helps children feel the safety and harmony of the family environment. Parents need to learn to perceive and adjust their emotions. For preschool children, when the child is crying and clings to others, parents could comfort them by touching and hugging, and could play games with them to relax and divert their attention; for school-age children and adolescents, parents need to listen patiently and accept their emotions when they are nervous. The attitudes of parental acceptance could help children restore calm. In contrast, if parents cannot listen patiently, but are eager to refute, reason, or even rebuke, this may only escalate the children’s negative emotions.

Receiving scientific and objective information can reduce children’s anxiety and promote their emotional stability. Many teenagers have access to relevant information but may be emotionally fluctuated by some misleading information on the internet. If they are too aggressive or indifferent, then parents should take actions to discuss and help them identify scientific and objective information by referring to official and authoritative sources. Paying more attention to the positive information in the news could increase the sense of hope.

The panic caused by the disease stems from the worst result it may cause; however, health care researchers and scientists from around the world have provided people with a large amount of scientifically feasible prevention knowledge and information [36,37]. Parents are supposed to work with children to develop disease prevention approaches based on their family’s characteristics. Children should be encouraged to communicate with friends and families by phone or internet, which could increase their sense of connection with the same situation—an effective psychological protection against the feeling of isolation and helplessness.

The COVID-19 pandemic has become a new normal around the world [38]. Quarantine and social distancing are becoming regular features of people’s daily life. Textbox 1 illustrates the negative influences of the long-term effects of physical and social isolation, and interventions for children.

**Impacts of the global public health emergency on children’s mental and behavioral health and possible interventions.**

**Loneliness and relevant sequelae [39]**

Using social media to bridge social distance [40,41]

**Distress [42]**

Open communication and discussions should be encouraged in case parents are underestimating children’s concerns [12,42].

**Anxiety, depression [43]**

Tracking and reducing sustained feelings of loneliness [44]

**Self-harm or suicide [45]**

Promoting children’s sense of belonging [46]

## Implications to Clinical Practice

The emerging technologies have been playing an increasingly more crucial role during the COVID-19 pandemic [47]. Providers should take timely actions to ensure children’s immediate health care needs are addressed, and children’s families are supposed to be actively engaged. Since face-to-face care has become less accessible, remote consultation and diagnosis are more suitable. Telehealth has the capacity and is appropriate for pediatric specialists to provide services during the pandemic, but health care providers need clear evidence-based guidelines to support children’s mental and behavioral health [48]. Children with mental illnesses are especially vulnerable during the quarantine and social distancing period. The inclusion of psychosocial support for children and their families are part of the health responses to disaster and disaster recovery. Pediatric clinical practices should reserve inpatient facilities for those children and adolescents for whom outpatient measures are not an adequate clinical option while using outpatient treatment options to the greatest extent possible. Adopting telehealth and telemedicine services enables the increased reliance on remote consultation and diagnosis, which further ramps up other necessary health care deliveries at children’s homes.

**Figure 1:**
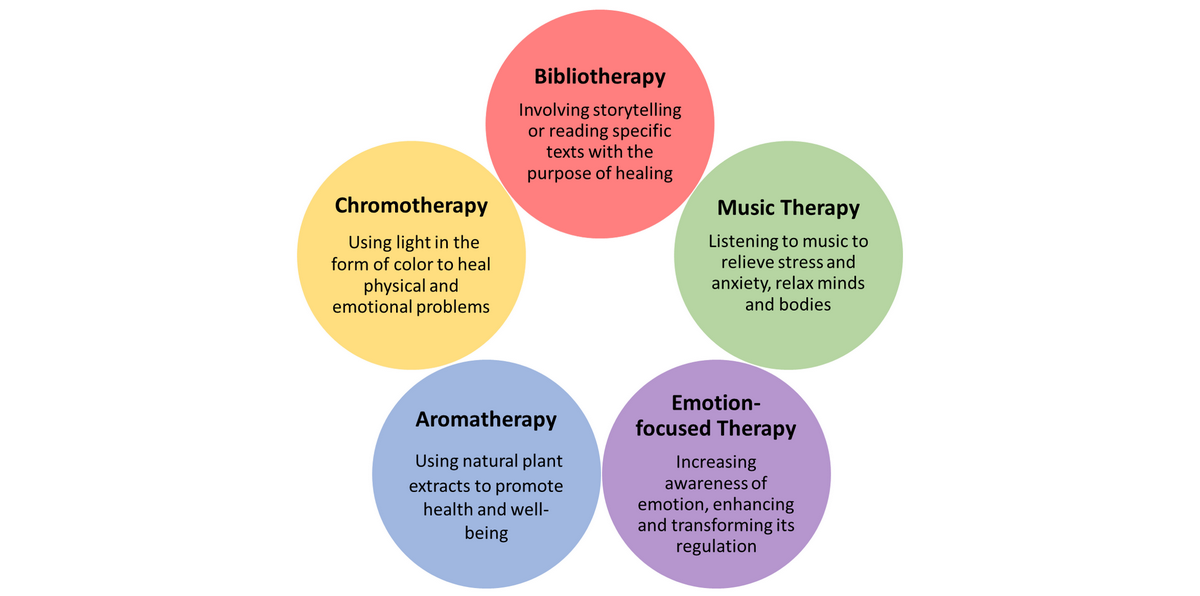
Psychological therapeutic interventions and approaches.

Meanwhile, psychological therapeutic practices could be alternative intervention approaches for children’s mental and behavioral health. Figure 1 demonstrates five common therapeutic approaches that could be applied to children with the guidance of health care providers and assistance from families.

Bibliotherapy involves storytelling and reading specific texts with the purpose of healing; through these cost-effective activities, children who have severe mental conditions could be adjusted to moderate or mild symptoms [49]. Chromotherapy leverages the color spectrum of light to balance energy on an emotional, physical, or spiritual level, thus aiding children with mental illnesses [50]. Children could also listen to music to promote wellness and manage stress, since this evidence-based intervention has been shown to improve emotional, cognitive, and communicative health and quality of life [51,52]. Additionally, with the guidance of health care professionals, parents may use aromatherapy to improve the physical health and spirits of children. Aromatherapy is a complementary treatment that uses essential oils medicinally to improve physical, psychological, or behavioral health [53]. Emotion-focused therapy (EFT) provides therapeutic approaches to connect parents and children, therefore improving problematic psychological states and interpersonal relationships. Meanwhile, EFT could consolidate the security gained through these new patterns of connection and restructure family’s interactions [54].

## Discussion

Families are the warmest havens for children. Parents are the closest supporters and protectors of children during the global pandemic. Maintaining close and open communication with children is the key to identifying their physical and mental health problems, and it provides corresponding actions and support. Psychological crisis interventions targeted to different psychological problems for different age groups should be conducted to reduce the psychological traumas and subsequent psychosocial problems caused by the pandemic. Communities and schools are playing unique and vital roles in supporting children by providing effective interventions with high efficacy. Parents and families should take more care of children’s mental health in their early life pathways, as good educational strategies are particularly important during the COVID-19 pandemic.

Even though the world has been struggling to curb the influences of the pandemic, the quarantine and social distancing policies will have long-term impacts on children. Innovative digital solutions and informatics tools are needed more than ever to support the health care systems, thus mitigating the negative consequences on children. Diverse works have been introduced and mobilized around the world. For instance, interactive data visualization tools have been used to display the pandemic information [55], mobile health apps have been used to track symptoms and contact tracing [56], and data-driven models and advanced algorithms have been employed to predict pandemic situations, which helps different parties and departments to make responses [47].

Future research on mental and behavioral health of pediatrics should pay more attention to novel solutions that incorporate interdisciplinary interactive technologies and digital approaches; leveraging considerable advances in pervasive or ubiquitous computing, human-computer interaction, and health informatics among many others. Health care delivery and services should envision and implement innovative paradigms to meet broad well-being needs and children’s health as the quarantine and social distancing over a longer term becomes a new reality [57]. Digital approaches, health technologies, and informatics are supposed to be designed and implemented to support public health surveillance and critical responses to children’s growth and development. For instance, human-computer interactions, augmented reality, and virtual reality could be incorporated to remote psychological supporting services for children’s health; mobile technologies could be used to monitor children’s mental and behavioral health while protecting their individual privacy; big data and artificial intelligence could be used to support decision-making on whether children should go out for physical activities and whether schools should be reopened.

### Conclusions

The physical and mental health of pediatrics directly affect their growth. Paying attention to the children and adolescents during the global public health emergency is of special social significance and clinical value for preventing the occurrence of mental disorders and adverse events. Humanistic care and psychological interventions for children should be included in the response strategies for the COVID-19 pandemic. The emerging digital applications and health services such as telehealth, social media, mobile health, and remote interactive online education are able to bridge the social distance and support mental and behavioral health for child populations. Health care delivery and services should envision and implement innovative paradigms to meet broad well-being needs and children’s health as the quarantine and social distancing over a longer term become a new reality. Digital approaches, health technologies, and informatics should be designed and implemented to support public health surveillance and critical responses to children’s growth and future development.

## Abbreviations

COVID-19: coronavirus disease
EFT: emotion-focused therapy
